# Knowledge and attitude on maternal health care among rural-to-urban migrant women in Shanghai, China

**DOI:** 10.1186/1472-6874-9-5

**Published:** 2009-03-20

**Authors:** Qi Zhao, Asli Kulane, Yi Gao, Biao Xu

**Affiliations:** 1 School of Public Health, Fudan University, Shanghai 200032, PR China; 2 Division of International Health (IHCAR), Karolinska Institutet, Stockholm, Sweden; 3 Nanhui District Center Hospital, Shanghai, 201300, PR China

## Abstract

**Background:**

In China, with the urbanization, women migrated from rural to big cities presented much higher maternal mortality rates than local residents. Health knowledge is one of the key factors enabling women to be aware of their rights and health status in order to seek appropriate health services. This study aims to assess the knowledge and attitude on maternal health care and the contributing factors to being knowledgeable among rural-to-urban migrant women in Shanghai.

**Methods:**

A cross-sectional study was conducted in a district center hospital in Shanghai where migrants gathered. Totally 475 rural-to-urban migrant pregnant women were interviewed and completed the self-administered questionnaire after obtaining informed consent.

**Results:**

The mean score of knowledge on maternal health care was 8.28 out of 12. However, only 36.6% women had attended the required 5 antenatal checks, and 58.3% of the subjects thought financial constrains being the main reason for not attending antenatal care. It was found that higher level of education (OR = 3.3, 95%CI: 1.8–3.8), husbands' Shanghai residence (OR = 4.0, 95%CI: 1.3–12.1) and better family income (OR = 3.3, 95%CI: 1.4–8.2) were associated with better knowledge.

**Conclusions:**

Rural-to-urban migrant women's unawareness of maternal health service, together with their vulnerable living status, influences their utilization of maternal health care. Tailored maternal health education and accessible services are in demands for this population.

## Background

China has gone through an extraordinary phase of development since 1978, however, the accelerated pace of industrialization and urbanization widened the economic gap between urban and rural areas [1,2]. Industrialization and the upgrading of China's economic structure have created a great number of job opportunities in urban areas, and released millions of rural laborers from agricultural production. As a result, the number of rural-to-urban migrants began to increase dramatically during the late 1980's and early 1990's. According to China National Census in 2000, the number of domestic migrant people in cities has reached 120 million with predictions of at least 160 million by 2010. Rural migrants are mostly concentrated in the booming cities of eastern China [3].

Shanghai, the financial and economic center of China, is the area with highest proportions for the cross-province immigrants [4]. There were only 600,000 non-Shanghai-residents in 1984, whereas in 2005 it had risen to 5,870,000, accounting for more than one-third of the total Shanghai population. Family reunification and labor migration are the major reasons for female migration to big cities. In Shanghai, 63.1% of the rural-to- urban migrants are at the age of 20–39 years with equally distribution of males and females [5].

The rural/urban designation of population is embodied in "hukou", a residence registry system of thousand years' history, which required every person registers in the same place under his/her parents' residence. Registered residents in the cities are entitled to many social benefits, such as medical insurance, pension, education, health services and other kind of welfare benefits, which rural to urban migrants are not eligible for. The system also keeps migrants out of stable employment in cities. The jobs that rural-to-urban migrants can find are often those considered as "dirty," high-risk or very low-paying [6]. They mainly undertake physical labor work in transportations, constructions, and services with much less payments compared to the registered residents [7]. Emigrated from poor and less developed rural provinces, the migrant workers are seen as uneducated, ignorant and low-class by many urban residents and considered as non-residents in the host city.

Mother and child health is clearly on the international agenda as a specific Millennium Development Goal. Over the last 20 years, China has made remarkable progresses in improving maternal health in both rural areas and urban cities. The maternal mortality rate (MMR), as an universal indicator, has plummeted from 94.7/10^5 ^in 1990 to 56.2/10^5 ^in 1998, and further to 36.6/10^5 ^in 2007 [8].

Analysis on maternal mortality in Beijing, Shanghai and Guangzhou from 2000 to 2002 showed that the maternal mortality in rural migrant women in these cities was significantly higher than that in city women. Moreover, deaths among rural migrant women were dominated by direct obstetric reasons, and most of the deaths were avoidable. In Shanghai, the MMR was 8.58/10^5 ^in 2005, comparable to MMR in high-income countries. However, when separated, the MMR was 1.64/10^5 ^for Shanghai residents and 48.46/10^5 ^for rural migrants [9]. Similar phenomenon occurs in other cities where a lot of migrant workers are located [10-12]. It is said that the interaction between health and migration is a complex and dynamic one that is influenced by the socio-economic and cultural background of migrants, their previous health history and experiences in access to health care, their knowledge and perceptions on health and their access to health care in the immigrated society [13].

Health knowledge is considered as one of the key factors that enable women to be aware of their rights and health status in order to seek appropriate health services. There are very few studies addressing rural-to-urban migrant women's knowledge and attitudes on maternal health care in China, and the association between socioeconomic and demographic factors and knowledge on maternal health care haven't be well concerned in this sub-population.

The objectives of the study are to assess the knowledge and attitude on maternal health care among rural-to-urban migrant women in Shanghai; to identify contributing factors to being knowledgeable on maternal health care, and to provide information for further maternal health education in rural-to-urban migrant women.

## Methods

### Study Setting

The study was carried out in a migrant gathering suburb district of Shanghai – Nanhui District. In 2006, the number of rural migrants was 202,500 constituting almost one third of the whole population in Nanhui district, of which 43.6% were female [14]. The number of births in local residents was 3590 with a birth rate of 4.94 per thousand in 2006 in Nanhui district, while it was 5566 and 14.15 per thousand respectively in rural-to-urban migrants.

Nanhui District Center Hospital is a secondary hospital serving for the district population. From July 2006 to June 2007, there were about 2000 births in Nanhui Center Hospital, which accounted for one out four births in Nanhui district, and among them 971 were by rural-to-urban migrant women. The other deliveries were distributed in the other two secondary hospitals and four primary hospitals in township.

### Study design

This was a cross-sectional study carried out in Nanhui District Center Hospital. The study subjects were rural-to-urban migrant women who didn't have a Shanghai residence (hukou), and who came to Nanhui District Center Hospital for delivery. Considering the seasonal variation, the subjects were recruited every other month from July 2006 to June 2007.

### Data collection

Subjects were invited for a self-administered questionnaire on knowledge (12 questions) and attitude (5 questions) of maternal health care. For the illiterate women, an ask-and-answer procedure was offered by an interviewer. The knowledge questionnaire was developed based on the routinely used materials of maternal health education in China. Information on demographics (age, emigrating place, duration in Shanghai, and husband's residence), socioeconomic status (education years, occupation of the couple and self-reported household income), and previous and current pregnancies of subjects were also collected using a structured questionnaire.

### Data Analysis

The database was built in EpiData version 3.1 for Chinese and the statistic analysis was carried out in SPSS 11.0.1 (Sn: 3805233, Chicago, IL, USA). Mean, median, quartiles and percentage were used in description. Answers to questions of knowledge were also scored as '1' for correct and '0' for wrong for each question. Student's t-test and ANOVA were used for comparing knowledge scores between subgroups at different demographics and socioeconomic status. Logistic regression was applied in analyzing the association between socio-demographics and knowledgeable level toward maternal health care.

### Ethic consideration

Informed consents were sought from all the participants after giving a description of the study prior to the interview. Approval for this study was obtained from the Ethics Committee of the School of Public Health, Fudan University

## Results

### General characteristics of subjects

None of the eligible women refuse to participation. In total, 475 rural migrant women from 21 of China's 31 provinces were recruited. Table 1 presents the general characteristics and pregnancy information of the subjects, and the husband's general information as well. The age of subjects in mean was 27-years-old ranged from 18–43 years. Of the 475 subjects, 7.6% were illiterate and 20.3% attended primary school only. The average duration in Shanghai was 3.07 years. Before the current pregnancy, 32.4% of the subjects were unemployed, 54.5% of them were labor workers. The average annual family income was 24734 CNY (1 USD = 7.5 CNY). Forty percent of these families had an annual income lower than 20000 CNY. Only 36.6% women have attended 5 or more antenatal checks required by the routine maternal health care, and 3.8% of the subjects didn't attend any antenatal checks (Table 1). The mean week of first antenatal check was at week 16 of gestation.

**Table 1:** The general demographics and socio-economic characteristics of subjects

Variable		No.	%
*Age groups (years)*	≤ 20	11	2.3
	20-	181	38.1
	25-	131	27.6
	30-	100	21.1
	≥ 35	52	10.9
*Duration of living in Shanghai (years)*	<1	108	22.7
	1-	138	29.1
	3-	105	22.1
	≥5	124	26.1
*Education*	Professional college or higher	9	1.9
	Senior high school (9~12 years)	41	8.7
	Junior high school (6~9 years)	291	61.5
	Primary school (≤ 6 years)	96	20.3
	Illiterate	36	7.6
*Occupation*	Non-labor worker	62	13.1
	Labor worker	259	54.5
	Unemployed	154	32.4
*Previous induced/spontaneous abortion*	No	284	59.8
	Yes	191	40.2
*Previous deliveries*	0	212	44.6
	1	216	45.5
	≥ 2	47	9.9
*Husband's residence*	Shanghai resident	39	8.2
	Non-Shanghai resident	436	91.8
*Husband's education*	Professional college or higher	20	4.2
	Senior high school (9~12 years)	80	16.8
	Junior high school (6~9 years)	303	63.8
	Primary school (≤ 6 years)	58	12.2
	Illiterate	14	2.9
*Husband's occupation*	Non-labor worker	123	25.9
	Labor worker	352	74.1
*Annual family Income (CNY)*	<20000	190	40.0
	20000-	236	49.7
	≥ 50000	49	10.3
*Antenatal care (times)*	0	18	3.8
	1-	283	59.6
	≥ 5	174	36.6

The mean of gestation week at delivery was 39.33 ranged from 28 to 45 weeks. Of the 475 women subjects, 33 (7.2%) had premature while 38 (7.6%) had postmature delivery. More than half (56%) women had obstetric complications including fetal distress, premature rupture of fetal membranes, prolonged pregnancy, abnormal fetal position etc. The mean birth weight of new born was 3341 grams, with a range of 1050 to 5000 grams. Fifty-two (10.9%) of the babies were giant whereas 21 (4.8%) were born with low-birth weight.

### Knowledge on maternal health

Table 2 shows the rural migrant women's responses to the questions on knowledge. Most of the subjects gave the correct answer to questions on common health knowledge, such as 'Is it needed to go to hospital when severe headache or vision problem happens?' In addition, they were very knowledgeable of breastfeeding (93.5%) and child immunization (95.8%). Although 71.2% agreed that antenatal care is necessary, more than half of the women didn't know the proper gestation months for first antenatal check. About 50.7% of them didn't know that anemia during pregnancy is preventable, and much fewer (28.8%) could correctly answer the question about the newborn deformity. For questions addressing necessity of fetal movement counting, calcium supply and blood pressure checking, and correct action after amniotic fluid breaks, 60–68% of the women got the correct answer. The mean score of knowledge on maternal health care was 8.28 out of 12, with the median 9, standard deviation 2.564 and quartile 7–10.

**Table 2:** Knowledge on maternal health care among the rural-to-urban migrant pregnant women

**Knowledge on maternal health care**	**No. of Subjects with correct answer**	**%**
Do you think antenatal care is needed?	338	71.2
Should first antenatal examination be done within the first 3 months?	208	43.8
Is it needed to count fetal movement everyday in the late stage of pregnancy?	287	60.4
Can anemia be prevented by eating more iron-contained food during pregnancy?	234	49.3
Does pregnant woman need calcium supply?	323	68.0
Should pregnant woman often check blood pressure?	318	66.9
What action should be taken after amniotic fluid breaks? (keep lying, keep sitting, no special attention, or unknown)	304	64.0
Is it needed to go to hospital when severe headache happens?	448	94.3
Is it needed to go to hospital when vision problem happens?	438	92.2
At which stage of pregnancy does newborn deformity most likely to happen? (<12 weeks, 12–28 weeks, >28 weeks, unknown)	137	28.8
Which method is better for feeding newborns (breast feeding vs. milk powder feeding)?	444	93.5
Should child be vaccinated?	455	95.8

### Attitude and willingness toward maternal health care

Of the participants, 58.3% thought the main reason for not attending antenatal care being financial constrains. Nearly 80% of the women wanted to get professional advices of infant feeding from health worker, and two-thirds wished to be visited by health workers during postpartum period. Regarding breastfeeding, 56.2% of the subjects indicated to breastfeed their baby for 4–10 months. About two in five of the women wanted to return to work within 2 months after delivery (Table 3).

**Table 3:** Attitude towards maternal health care among the non-resident migrant pregnant women

**Question**	**answer**	**%**
*What is the main reason for not attending antenatal care as required?*		
No time	55	11.6
Transportation inconvenience	36	7.6
Financial difficulties	277	58.3
No need	45	9.5
Physician's bad attitude	15	3.2
Others	47	9.7
*Would you like to get instruction of infant feeding from health worker?*		
Yes	374	78.7
Doesn't matter	58	12.2
No	43	9.1
*Would you like to be visited by health worker during postpartum period?*		
Yes	312	65.7
Doesn't matter	93	19.6
No	70	14.7
*How long would you like to breastfeed?*		
0–3 months	38	8.0
4–10 months	267	56.2
11 months and above	170	35.8
*When would you like to return to work after giving birth?*		
0–2 months	191	40.2
3–6 months	130	27.4
7 months and above	154	32.4

### Distribution of knowledge score and it's contributing factors

Scores of knowledge were compared between women at different age groups and with different socioeconomic status. It was found that younger women, women and their husbands having better education, being non-labor workers, primiparas, having a Shanghai husband, and having better family income had significantly higher knowledge scores (Table 4). In addition, women who have attended 5 times or more antenatal care had a statistically significant higher knowledge score than those who had less antenatal visits (9.45 ± 2.17 vs. 7.60 ± 2.53, t = 8.070, P < 0.001). There were no significant differences in knowledge score between women with and without obstetric complications; and women having a baby at low-birth-weight or not (P > 0.05).

**Table 4:** Knowledge scores on maternal health care in rural-to-urban migrant pregnant women at different demographics and socioeconomic status

**Variable**		**No**.	**M ± s**	**Statistical results**	
***Age groups (yrs)***	<25	192	8.49 ± 2.48	*F *= 10.101	***P *< 0.001**
	25~	131	8.98 ± 2.31		
	30~	100	7.54 ± 2.61		
	≥35	52	7.19 ± 2.76		
***Duration in Shanghai (yrs)***	<3	246	8.14 ± 2.42	*t *= 1.233	*P *= 0.218
	≥3	229	8.43 ± 2.70		
***Education (yrs)***	≤ 6	132	6.72 ± 2.33	*t *= 8.891	***P *< 0.001**
	>6	343	8.89 ± 2.39		
***Occupation***	Unemployed	154	7.79 ± 2.63	*F *= 7.283	***P *= 0.001**
	Non-labor worker	62	9.22 ± 2.56		
	Labor worker	259	8.35 ± 2.45		
***Previous deliveries ***	Primipara	212	8.94 ± 2.46	*t *= 5.222	***P *< 0.001**
	Multipara	263	7.74 ± 2.52		
***Previous abortion***	no	284	8.19 ± 2.57	*t *= 0.990	*P *= 0.323
	yes	191	8.42 ± 2.56		
***Husband's residence***	Shanghai	39	10.28 ± 2.02	*t *= 5.223	***P *< 0.001**
	Non-Shanghai	436	8.11 ± 2.53		
***Husband's education (yrs)***	≤ 6	72	6.47 ± 2.16	*t *= 6.806	***P *< 0.001**
	>6	403	8.61 ± 2.50		
***Husband's occupation***	Non-labor worker	123	9.07 ± 2.42	*t *= 3.996	***P *< 0.001**
	labor worker	352	8.01 ± 2.56		
***Annual family income (CNY)***	<20000	189	7.38 ± 2.66	*F *= 29.006	***P *< 0.001**
	20000-	237	8.62 ± 2.36		
	≥ 50000	49	10.10 ± 1.54		
***Antenatal care visit (times)***	<5	301	7.60 ± 2.53	*t *= 8.070	***P *< 0.001**
	≥ 5	174	9.45 ± 2.17		

Multivariate analysis was applied using Logistic regression with the mean of knowledge score (8.28) as dependent variable (1 for score >= 8.28, and 0 for score <8.28). It was found that women had a husband with Shanghai residence and had education of junior high school or higher was associated with a better knowledge level (OR = 4.0, 95% CI: 1.3–12.0, and OR = 3.3, 95% CI: 1.8–5.8 respectively). Women at highest family income were likely to have a better knowledge level on maternal health care (OR = 3.3, 95% CI: 1.4–8.2) (Table 5).

**Table 5:** Logistic regression on factors associated with knowledge level of maternal health care in rural-to-urban migrant pregnant women

***Variable***		***χ***^**2**^	***P***	**OR**	**95% CI**	
***Age groups (yrs)***	≥ 35			1.000		
	30~	0.209	0.647	0.812	0.332	1.984
	25~	0.022	0.881	1.064	0.471	2.404
	<25	0.231	0.631	0.819	0.362	1.852
***Education (yrs)***	≤ 6			1.000		
	>6	15.670	**<0.001**	3.254	1.814	5.837
***Occupation***	Labor worker			1.000		
	Unemployed	0.667	0.414	0.821	0.511	1.318
	Non-labor worker	0.179	0.672	1.185	0.541	2.594
***Duration in Shanghai (yrs)***	<3			1.000		
	≥ 3	0.031	0.860	0.961	0.616	1.499
***Previous abortion***	Yes			1.000		
	No	1.431	0.232	0.764	0.492	1.187
***Previous delivery***	Primipara			1.000		
	Multipara	3.566	0.059	0.662	0.431	1.016
***Husband's residence***	Non-resident			1.000		
	Shanghai resident	6.146	**0.013**	4.021	1.338	12.083
***Husband's education***	≤ 6			1.000		
	>6	3.002	0.083	1.859	0.922	3.750
***Husband's occupation***	Labor worker			1.000		
	Non-labor worker	1.061	0.303	1.343	0.766	2.352
***Annual family income (CNY)***	<20000			1.000		
	20000-	2.479	0.115	1.430	0.916	2.233
	≥ 50000	6.934	**0.008**	3.343	1.361	8.209

## Discussion

### Methodological consideration

In this hospital-based cross-sectional study, all the subjects were continuously recruited when they came for delivery in the study period. These subjects could be good representatives to rural-to-urban migrant women who delivered in hospitals, but not to those who go back their hometown for delivery or give a birth outside the legal maternal health facilities. The knowledge questions used in this study were extracted from the widely-used maternal health education materials in China.

### Knowledge on maternal health care

A study in rural Guatemala found financial cost and geographic access were the most important barriers to formal delivery assistance, whereas awareness and acceptance remain as important barriers to the use of formal prenatal care [15]. Similar study showed rising the awareness of women about reproductive health may improve the women's understanding of their own reproductive health and contribute to their acceptance and utilization of available reproductive health services [16].

Findings of this study showed that most of the migrant women knew the necessities of seeking antenatal care. However, a majority of them didn't know first care-seeking should be done within the first trimester of pregnancy. A large number of women subjects did not know how to handle possible urgent problem at home, almost 40 percent of them didn't know correct actions after amniotic fluid breaks. Nearly half of the participants did not know the effects of iron-rich food on preventing anemia. Only few of the rural migrant women knew the key stage of developing deformity, let alone how to prevent birth defects.

It is well known that health education on maternal care be mainly provided to the pregnant women by health workers at the time of antenatal examination. Xue described maternal health knowledge level was higher among the pregnant women who attended antenatal care than those who did not seek antenatal care [17]. Migrant women's lack of maternal health care knowledge may be due to not attending antenatal care and/or insufficient information received at the antenatal care.

Optimal breastfeeding practices include exclusive breastfeeding (breast milk with no other foods or liquids) for the first six months of life, and continued breastfeeding for up to at least two years of age while receiving complementary foods [18]. In China, breastfeeding rates varied in different parts of area considerably. In rural areas 91.2% of the women did exclusive breast-feed for 4 to 6 months compared to 44.4% of urban women [19]. This can be mostly attributed to the traditions in rural China, which encourage exclusive breastfeeding. However, reasons for breastfeeding pattern were not measured in this study.

Expanded Program of Immunization in children has been implemented on a nationwide scale in China since 1978, thus it is not difficult to understand that most of migrant women subjects perceived the necessity of immunization to their babies.

### Factors having impact on knowledge level

An important finding in this study was that the socio-economic status of education, husband's residence and annual family income, and delivery experiences were the main factors influencing the knowledge level of maternal health care among rural migrant women.

Education was found to have the most powerful influence on the knowledge score of maternal health. Knowledge not only transforms, but also empowers women and improves their self-esteem [20]. It is expected that educated women are more likely to aware their health status and seek health knowledge. Furthermore, educated women may have a greater decision making power on health related matters. At present, in China, a large number of rural-to-urban migrant women have very limited education, and it is difficult for them to access maternal health knowledge. This could lead to inadequacy utilization of maternal health care service [21,22]. A study in Paraguay has reported that maternal health knowledge would be improved with wide application of community-based antenatal care program to meet the needs of those who are functionally illiterate [23]. Considering the limited education in rural migrant women in urban cities, education program of maternal health carried out in migrant women gathered places including suburb communities, labor-density factories and serviced are in great demands.

Migrants' health outcomes were associated with their language skills and familiarity with the culture of the host community [13]. The circumstances surrounding the migration itself as well as the social and health characteristics of re-settlement were also found to influence the outcomes [24]. In our study, women having a Shanghai-resident husband had better knowledge on maternal health. The stable employment, the obliged 9 years education and the comprehensive medical insurance for Shanghai residents give the husbands better access to maternal care for their rural migrant wives.

We found a considerable number of women in our study were unemployed and the families were living in a low-income status. Poor women usually have poor access to education, including health education due to lack of financial resources, early marriage and pregnancy, household responsibilities and unwillingness to invest in the hidden costs of education (fees, transport, etc). It is obvious that women with least knowledge on maternal health would be the one who had least access to maternal health care services.

Bogg's study has reported that financial difficulties became the most important reason for not seeking health care for people with low income in China [25]. Our study also found that almost 60 percent of the participants thought the main reason for not attending antenatal care being financial difficulties. In Shanghai, migrant women who are not permanent Shanghai residents are not covered by the municipal social-medical insurance. Thus, the poor migrant women are the vulnerable population in need of access to maternal care.

An interesting finding in this study is that the multiparaes had a poorer knowledge on maternal health than the primiparaes although it was not statistically significant after adjusting with demographics and other socio-economic factors (*P *= 0.059). Rural migrant women with limited education are more likely to get knowledge of pregnancy from friends and relatives; meanwhile, their experience of pregnancy and delivery made them believe their maternal health knowledge was adequate. However, their 'knowledge' might only be traditional beliefs and habits. Several studies have reported that the IMR increases with the order of birth [26,27]. The outcome of maternal was closely associated with birth order [28,29].

## Conclusions

In conclusion, this study reported incomprehensive knowledge on maternal health care among rural-to-urban migrant women in Shanghai. Antenatal care attendance was constrained by financial difficulties. Woman's education level, husband's urban residence and family income were showed to be important factors influencing maternal health knowledge. Findings from this study suggest the need for targeted health education using various educational methods for rural migrant women, who are vulnerable group living in urban cities. Healthcare providers, educators and policy makers can use these insights, to develop strategies and further investigation assessing the health service needs of rural-to-urban migrant pregnancy women.

## Competing interests

The authors declare that they have no competing interests.

## Authors' contributions

QZ participated in data analysis and wrote the manuscript. BX conceived the idea, implemented the field study and wrote the manuscript. YG participated in the design and implement of the study and statistical analysis. AK participated in data analysis and helped to draft the manuscript. All authors read and approved the final manuscript.

## Pre-publication history

The pre-publication history for this paper can be accessed here:


